# Pleural Space Infections

**DOI:** 10.3390/life13020376

**Published:** 2023-01-29

**Authors:** Sean P. F. Foley, John Scott Parrish

**Affiliations:** Department of Pulmonary, Critical Care and Sleep Medicine, Naval Medical Center San Diego, San Diego, CA 92134, USA

**Keywords:** pleural infections, pleural disease, empyema, pneumonia, pleural effusion, parapneumonic effusion, pleural sepsis, pulmonary infections, interventional pulmonology, unilateral pleural effusion

## Abstract

Pleural space infections have been a well-recognized clinical syndrome for over 4000 years and continue to cause significant morbidity and mortality worldwide. However, our collective understanding of the causative pathophysiology has greatly expanded over the last few decades, as have our treatment options. The aim of this paper is to review recent updates in our understanding of this troublesome disease and to provide updates on established and emerging treatment modalities for patients suffering from pleural space infections. With that, we present a review and discussion synthesizing the recent pertinent literature surrounding the history, epidemiology, pathophysiology, diagnosis, and management of these challenging infections.

## 1. Introduction

Pleural space infections have been recognized as a cause of disease since at least 3000 BC when first described by the Egyptian physician Imhotep [1]. However, it was the Greek physician Hippocrates who, recognizing the severity of these infections, first attempted drainage of the pleural cavity over 2000 years ago [2]. Despite these early attempts at treating empyema, mortality remained staggeringly high and, in the early twentieth century, was conservatively reported to be between 20 and 30% and as high as 70% in military camps where early open drainage was standard. Based upon the staggeringly high mortality associated with empyema during the 1918 flu pandemic, the Surgeon General created the Army Empyema Commission. The work of this group led to significant advances in the care of patients with pleural space infections. Among the efforts of the commission members, perhaps most notable were those of United States Army Surgeon Dr. Evarts A Graham, who published “Some Fundamental Considerations in the Treatment of Empyema Thoracis” in 1925 [3]. 

Predating antibiotic therapy, much of Dr. Graham’s work focused on the procedural aspects of empyema management along with the importance of patient nutrition and support. Included was an extensively detailed analysis of the basic principles of lung mechanics, pleural dynamics, and ventilatory work with a specific discussion surrounding patient factors that were favorable for the performance and successful outcome of a thoracotomy. He estimated the maximum opening of the pleural cavity that was compatible with life (estimated to be 5 cm by 10 cm) and specifically detailed factors such as increased respiratory demand, decreased alveolar ventilation due to pneumonia or mucus plugging, and weakened respiratory muscles, all of which would decrease the potential size of thoracotomy. Ultimately, he concluded that since all the above factors are likely to be present in the early stages of an empyema, early operation with the establishment of open pneumothorax carries “unwarrantable danger”, with Dr. Graham and the Army Commission settling on the following principles to advance empyema management. (1.) Careful avoidance of open pneumothorax in the acute stage, (2.) Prevention of chronic empyema by rapid sterilization and obliteration of the infected cavity, and (3.) Careful attention to the nutrition of the patient. With the widespread application of these principles, mortality from empyema fell to 3.4% [3].

The next advancement in empyema care involved the introduction of antibiotics, which reduced the incidence of empyema and changed its bacteriology [1]. Prior to antibiotic use, empyema was a complication in approximately 5% of cases of pneumonia; however, with the widespread introduction of antibiotics in the 1940s, the incidence of empyema decreased to approximately 2% of cases [4]. Concurrent with this, intrapleural fibrinolytic therapy became an area of active research that is still ongoing 75 years later. Subsequent advances in surgical techniques resulted in expanded procedural options to include Video Assisted Thoracoscopic Surgery (VATS) and Medical Thoracoscopy, with a corresponding decrease in the use of traditional thoracotomy [5]. 

## 2. Nomenclature of Pleural Fluid in Pleural Infections

Despite having been recognized as a source of disease for millennia, the nomenclature surrounding pleural space infections can be a source of confusion among clinicians. Thus, a comprehensive understanding of the correct definitions of various pleural space conditions is essential for the proper diagnosis and management of pleural space infections; they are briefly reviewed here (Table 1). Pleural infection is defined as the entry and replication of a pathogenic organism in the pleural space. Bacterial sources of pleural infection are the most common and are the focus of this paper; however, pleural infections can be caused by certain fungal organisms as well. Pleural infection, as a general description, acknowledges the important observation that an associated pneumonia is not required and may be absent. 

A parapneumonic effusion is any pleural effusion associated with pneumonia or a lung abscess [6]. An uncomplicated parapneumonic effusion is generally free-flowing and will resolve with only antibiotics without requiring mechanical intervention [7]. A parapneumonic effusion becomes complicated when mechanical drainage is required for resolution (and is therefore referred to as a complicated parapneumonic effusion). There are several indicators in pleural fluid analysis that, if present, portend a low likelihood of resolution without mechanical drainage and thus should prompt tube thoracotomy. Classically, these are a pleural fluid pH < 7.20, pleural fluid glucose <40 mg/dL, pleural fluid LDH >1000 IU/L, or positive bacterial studies (Gram stain or culture) [1], [6,7,8,9]. However, it is important to note that only positive Gram stains and cultures are specific for pleural infection and that other non-infectious diseases may result in a similar pleural fluid biochemical profile; thus, the clinical context is important to consider as well. 

It is also important to note that the term “complex parapneumonic effusion” is often used synonymously with “complicated parapneumonic effusion”, and these terms are often used interchangeably in the medical literature. However, to avoid confusion, the descriptor “complex” may be better used as a physical description of the septations and loculations present within a pleural effusion (a “complex pleural effusion”) as opposed to the clinical infectious syndrome (a “complicated parapneumonic effusion”), as these complex septations and loculations can occur in other non-infectious pleural diseases as well. The terms “complicated parapneumonic effusion” and “complicated pleural infection” are used preferentially throughout this review to avoid confusion unless referencing a radiographic description of the pleural space, which may be described as a “complex pleural effusion”. Empyema is defined as pus in the pleural space (described as thick, white-yellow, viscous fluid resulting from serum coagulation proteins, cellular debris, and fibrin deposition) or pleural fluid with a positive Gram stain or culture [6,7]. 

## 3. The Pathophysiology of Pleural Space Infections

Empyema thoracis, which is Greek for “pus in the chest”, can result from infections in a multitude of different parts of the body that eventually infect the pleural space. However, the most common precursor is generally bacterial pneumonia and subsequent parapneumonic effusion. Other, less frequent, causes of empyema are associated with bronchogenic carcinoma, esophageal rupture, blunt and/or penetrating trauma of the chest, infectious mediastinitis with extension into the adjacent pleura, extension of infection across the diaphragm from intraabdominal sources, cervical or thoracic spine infections, and post-surgical infections [4].

The exact mechanism of pleural space infection likely results from increased permeability of the mesothelial layer of the inflamed pleura, allowing the invasion of bacteria into the otherwise sterile pleural space. It is notable, however, that a lack of radiographic evidence of pneumonia has been reported in 56% of community-acquired and 73% of hospital-acquired pleural space infections, and different microbial profiles have been observed between pneumonia and pleural space infections as well, providing potential support for hematogenous seeding as a mechanism for development in certain cases [10]. Despite the multifactorial mechanisms by which pleural space infections develop, the most common route seems to be secondary to aspiration of organisms from the oropharynx with subsequent development of pneumonia in the dependent lobes, which, if untreated, can progress to parapneumonic effusion and eventually empyema [7].

Classically, pleural space infections have been described as passing through several distinct phases which exist in a continuous spectrum. The first phase is characterized by an exudative effusion that results from a rapid outpouring of fluid into the pleural space. In this first phase, the pleural fluid will typically be culture and Gram-stain-negative, will have a glucose level greater than 60 mg/dL, and will have lactate dehydrogenase levels less than three times the upper limit of normal for serum [6]. Proinflammatory mediators such as TNFa, IL-6, and IL-8 are thought to play a prominent role in this phase [1,10].

The second phase is the fibrinopurulent stage which follows the initial exudative phase and may result if antibiotic treatment is inadequate or delayed. This phase is characterized by bacterial invasion into the pleural space [6,10]. In this stage, the pleural fluid will generally have a glucose level below 60 ng/mL with a pH below 7.20 and a pleural fluid LDH greater than three times the upper limit of normal [6]. Increased levels of plasminogen-activator inhibitors and TNFa lead to fibrin deposition forming septations and loculations. Within these loculations, walled-off bacteria cause increased phagocytic activity by neutrophils, a corresponding increase in LDH, and increased production of lactic acid and consumption of glucose, explaining the changes observed in pleural fluid analysis [10].

**Figure 1:**
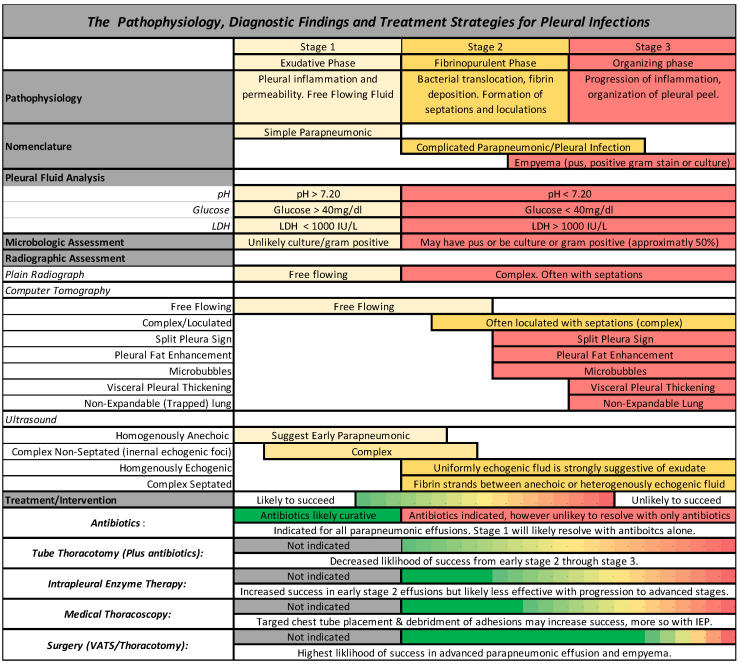
A schematic representation of the pathophysiology, diagnostic findings, and management strategies for pleural space infections.

The third and final phase is the organizing phase. This phase is characterized by fibroblast growth into the visceral and parietal pleura with deposition of a collagen-rich fibrin matrix in the pleural space and is accordingly characterized by pleural thickening. Formation of this inelastic visceral pleural peel often leads to lung entrapment. The fibroblast proliferation is thought to be due to the release of Transforming Growth Factor-Beta and Platelet Derived Growth Factor [6,10]. The stages of parapneumonic effusion, pleural infection, and empyema, along with the associated pleural fluid analysis, radiographic findings, and treatments, are graphically depicted in Figure 1. 

## 4. The Epidemiology of Pleural Space Infections

Despite recent advances in medical care, the incidence of pleural space infections has steadily increased over the past two decades [11,12,13,14,15]. Between 2005 and 2014, it is estimated that 150,469 adult patients (age > 18) were admitted to United States hospitals for pleural infections. The mean age of these patients was 58.3 years, 68% were males, 79.3% of patients admitted were Caucasian, and 4.4% of patients presented with severe sepsis (a category of sepsis abandoned in the Third International Consensus Definitions for Sepsis and Septic Shock), or septic shock [5].

Despite these staggering figures, data regarding hospitalization for pleural space infection are conflicting, as Gupta et al. noted a decrease in hospitalizations between 2005 and 2014 (decreased from 54.4 to 41.2 hospitalizations per million persons), with Mummadi et al. noting an increase from 2007 to 2016 [5,11]. Mortality data are also conflicting, as Gupta et al. noted a decrease from 4.2% to 2.6% between 2005 and 2014, with Mummadi et al. noting in-hospital mortality rates of 5.2–6.2% between 2007 and 2016 [5,11]. Despite the differing data on hospitalizations and mortality, there is a consensus that pleural space infections represent a major cost to the healthcare system. Inflation-adjusted costs per case were between USD 29,458 and USD 32,829 between 2005 and 2014 per Gupta et al. (adjusted to the 2019 Consumer Price Index), with the average cost per empyema being USD 38,591 per Mummadi et al. [5,11]. 

From a management perspective, data analysis from 2005 to 2014 is significant for several notable trends. Approximately 76.3% of patients with pleural space infections underwent either tube thoracostomy, Video Assisted Thoracic Surgery (VATS), or thoracotomy. Over the period, the use of tube thoracostomy as a first management option increased from 21.7% to 29.6%. VATS, as a procedure of first choice, increased from 11.2% to 14.9%, with a corresponding fall in the use of thoracotomy from 22.6% to 18.9%. It is also notable that 64.9% of patients who underwent tube thoracostomy as a first procedure did not require any additional procedures [5].

## 5. The Microbiology of Pleural Space Infections

Prior to the introduction of antibiotics, most pleural infections were caused by Streptococcus pneumonia (60–70%), followed by Streptococcus pyogenes and Staphylococcus aureus [16]. However, the introduction of antibiotics has caused a shift in the microbiology of pleural infections. A recent systematic review by Hassan and colleagues examined 6202 bacterial isolates in adults and showed that Staphylococcus aureus was the most frequently isolated organism (20.7%). Following this, the most isolated organisms were Viridians streptococci group (18.7%), Pseudomonas species (17.6%), Enterobacteriaceae group (11.9%), Strep pneumoniae (10.8%), Klebsiella species (10.7%), Acinetobacter species (5%), and coagulase-negative staphylococci (4.5%) [17]. In all, 50.4% of cases were Gram-positive aerobic organisms, 37.5% were Gram-negative aerobic organisms, and 12.1% were anaerobic organisms. Additionally, 12.9% of specimens were polymicrobial in nature, 75% of which involved an anaerobic bacterium mixed with other aerobic or anaerobic organisms [17]. It is also notable that bacterial cultures of pleural fluid were only positive in 56% of cases in this analysis, which is in line with data from other studies, indicating that in over half of all cases, no definitive organism will be isolated [17,18]. 

Hassan and colleagues also examined geographic differences in the microbiology of pleural infections based on latitude. In this analysis, isolates from the subtropical region (defined as latitudes between 23.5 and 40 degrees north or south) had a higher incidence of Gram-negative organisms in comparison to either the tropics (within 23.5 degrees latitude of the equator) or temperate regions (greater than 40 degrees north or south latitude) [17]. 

The microbiologic aspects of pleural infections are also notable, as different organisms have been associated with varying mortality. Specifically, an 83% survival rate was observed in patients with streptococcal isolates, similar to an 87% survival rate in patients who did not have an identifiable organism. With this, it is postulated that many culture-negative cases may be streptococcal in origin with prompt suppression of bacterial numbers following administration of antibiotics leading to culture negativity. Outcomes for anaerobic pleural infections were also favorable, with an observed 80% survival rate [19]. Contrasting this, infections caused by Staphylococcus, Enterobacter, and mixed aerobic organisms were observed to have significantly worse mortality, with only 55% survival observed at one year [19]. 

Data also show that hospital-acquired infections have a significantly increased risk of mortality with a one-year mortality rate of 47% as compared with community-acquired infections, which had a one-year mortality of 17% (RR 4.24, confidence interval 2.07–8.69, *p* < 0.00001) [19]. The most frequent isolates in hospital-acquired infections were Staphylococcus (25%), Enterobacteriaceae (18%), Pseudomonas (5%), and enterococcus (12%). Specific to Staphylococcus, 70% of isolates were methicillin-resistant. 

## 6. Clinical Presentation of Pleural Space Infections

Overall, 20–40% of patients hospitalized with pneumonia will have associated parapneumonic effusion. Of those, 5–10% with parapneumonic effusion will progress to empyema, with 30% of patients with empyema requiring surgical drainage and 15% of patients with empyema ultimately dying [4]. Considering this, prompt and accurate diagnosis and management are paramount to good patient outcomes, and any pleural effusion should be investigated as a source of infection in patients presenting with pneumonia or unexplained sepsis, especially those who do not respond clinically to appropriate antibiotic therapy within a few days of initiation. 

Unfortunately, presenting symptoms in patients with pleural space infection are not remarkably different from those in patients with pneumonia and uncomplicated parapneumonic effusion and may include fever, cough, malaise, dyspnea, and pleuritic chest pain [7]. On this basis, it is impossible to predict by history alone which patients will have an uncomplicated parapneumonic effusion that will resolve with conservative therapy vs. those with complicated parapneumonic effusions or empyema which will require mechanical drainage. Additionally, the presence of these constitutional symptoms in the presence of a unilateral effusion may also mimic malignancy.

## 7. Radiographic Investigation of Parapneumonic Effusion

Plain chest radiographs are commonly the first imaging obtained in patients presenting with respiratory illness and, thus, are generally the first studies that identify the presence of parapneumonic effusion. However, one of the ultimate questions regarding a parapneumonic effusion is whether thoracentesis should be performed and whether eventual mechanical drainage or surgery will be required. While much of this is ultimately guided by biochemical analysis of pleural fluid, radiographic investigation can play a role in selecting patients who require thoracentesis and in predicting those who may ultimately have a complicated course requiring further drainage. 

While not robustly defined in the guidelines, most recommendations are that diagnostic thoracentesis should be performed if feasible on all parapneumonic effusions with a pleural fluid thickness of >1 cm on a lateral decubitus chest X-ray or >2.0 to 2.5 cm on chest CT, as effusions smaller than this on CT have been shown to have a low likelihood of being complicated [20,21]. Subsequent decisions for pleural drainage should then be based on the macroscopic appearance and biochemical analysis of the pleural fluid [4]. 

Lateral decubitus films were traditionally used to identify which parapneumonic effusions required thoracentesis and merit discussion for their historical role in management. Light et al. initially advocated in 1980 that lateral decubitus films be obtained in all patients who had evidence of pleural fluid in the posterior costophrenic angle on lateral chest X-ray and that thoracentesis should be performed in all patients who subsequently had a pleural fluid thickness >10 mm on their lateral decubitus film. In their original study, thoracentesis was performed in 37 of 90 patients who met this criterion, 10 of which ultimately had a complicated parapneumonic effusion by biochemical analysis [22]. However, the recent availability of bedside ultrasound has obviated the need for lateral decubitus films, as ultrasound has been shown to be significantly more sensitive for the detection of pleural effusion [23].

**Figure 2:**
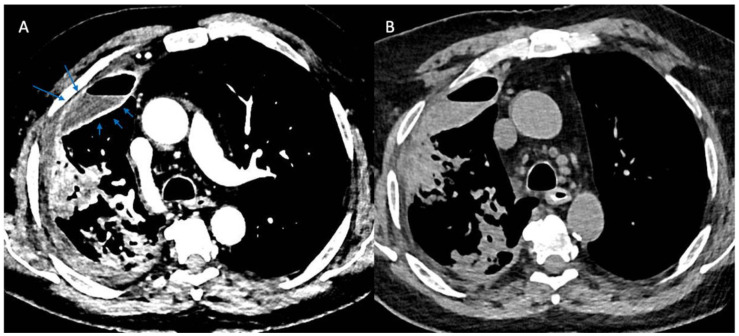
Split Pleura Sign. Panel (**A**) shows contrast enhancement of both the visceral and parietal pleura (arrows) in a patient with empyema (windowing optimized to show pleural enhancement). Panel (**B**) shows a non-contrast study in the same patient several days prior. Comparison of the two images demonstrates the value of contrast when evaluating patients with suspected pleural space infections.

More recently, computed tomographic imaging has proven useful in the radiographic assessment of empyema as it allows for a more thorough investigation of lung parenchyma, pleura, and extrapleural fat abnormalities. The organizing phase of complicated pleural infections is characterized by fibrin deposition on the visceral and parietal pleura with associated ingrowth of capillaries and fibroblasts. This forms the basis for pleural thickening and the split pleura sign (Figure 2) that was originally reported in 68% of CT scans in patients with empyema and was later found to be present in 98.7% of patients with pleural infection who underwent pleural phase contrast-enhanced CT [24], [25]. Furthermore, the presence of a split pleura sign with a total pleural fluid thickness of >30 mm was found to be 79.4% sensitive and 80.9% specific for distinguishing complicated parapneumonic effusion and empyema from non-complicated parapneumonic effusion [26]. More recently, Porcel et al. used multivariate regression analysis to generate a simple scoring system for patients with pleural infection who underwent pleural enhanced CT scan and found that it was also useful in separating non-complicated parapneumonic effusions from complicated parapneumonic effusions that required mechanical drainage. Specifically, a total of four points when combining the presence or absence of pleural contrast enhancement (3 points), pleural microbubbles (1 point), increased attenuation of extrapleural fat (1 point), and pleural fluid volume ≥ 400 mL (1 point) were shown to be 84% sensitive and 75% specific with a positive and negative likelihood ratio of 3.4 and 0.22, respectively, for differentiating complicated from non-complicated parapneumonic effusions [27]. 

While many pleural effusions are initially recognized on plain radiographs and CT scans, ultrasound has evolved as perhaps the most useful modality to aid in diagnosis and procedural planning. When studied previously, the presence of internal septations and an echogenic appearance of pleural fluid predicted an exudate with 100% accuracy (148/148 cases). However, these findings were not specific to pleural infection vs. alternative exudative causes [28,29]. Later, Svigals et al. similarly concluded that the above criteria were 69.2% sensitive but 90.0% specific for a complicated parapneumonic effusion (as defined by pleural fluid pH < 7.20, glucose < 60 mg/dL or positive Gram stain or culture) [30]. Additionally, when examining ultrasonographic pleural fluid patterns, Chen et al. found that anechoic fluid, homogenously echogenic fluid, or complex fluid without septations were all significantly more likely to resolve with only tube thoracotomy as compared with those effusions that had a complex appearance with septations (80.0% vs. 50.6%, *p* = 0.001). The latter group was also noted to have a statistically significant increase in mortality as compared with the former (21% vs. 7%, *p* = 0.018) [31]. However, it is also worth noting that septations and echogenicity of pleural fluid have not reliably been shown to correlate with the stage of parapneumonic effusion or predict the subsequent need for surgical treatment [32]. 

## 8. Prediction Models for Pleural Effusion

Rahman et al. recently devised the RAPID score with the goal of identifying patients with pleural infections who are at risk of poor outcomes. This score is a simple and easy-to-apply points-based score utilizing several readily available patient factors. Specifically, the patient’s renal function, age, characterization of pleural fluid, and setting of infection (hospital vs. community) are combined to generate the score [33]. This tool was initially derived from the MIST1 and MIST2 studies but has recently been validated by other investigators in retrospective cohort and prospective observational cohort analyses [34,35,36].

While the RAPID score has been shown to accurately predict mortality in pleural infections, its impact on management is less clear as it has not yet been proven useful in guiding procedural management. However, as a mortality prediction tool, it has allowed the comparison of cohorts and their ultimate outcomes and interventions in a standardized fashion. Corcoran et al. found that surgical intervention was highest in patients with low-risk RAPID scores (19.1% of patients received surgery) as compared with the high-risk group (5.9% of patients received surgery). Additionally, of the patients with a high-risk rapid score who failed medical therapy and, thus, would optimally have proceeded to surgery, only one in three ultimately received surgery [35]. This is in line with findings from other studies, which suggest that there is a selection bias for surgical candidates with improved baseline health. Specifically, Shin and colleagues noted that patients undergoing surgery, compared to those managed medically, had a more favorable BMI, better performance status, fewer comorbidities, and lower initial APACHE II scores [37]. 

While the RAPID score has not yet shown utility in selecting patients for certain management strategies, several alternative findings have been associated with the eventual need for surgery. Specifically, using multivariable regression analysis, Chang et al. identified the presence of pleuritic chest pain, white blood cell counts greater than 13,500 cells/uL, pleural loculations, and split pleura sign on contrast-enhanced CT to all be predictive of eventual surgery [38].

## 9. Pleural Fluid Drainage and Analysis

As described above, diagnostic thoracentesis should be performed when feasible in all patients who present with parapneumonic effusion. However, the decision to pursue diagnostic thoracentesis is often guided by initial radiographic imaging, and unfortunately, there is little high-quality data regarding which radiographic findings will predict or exclude a complicated parapneumonic effusion and its consequent clinical course. 

Traditionally, pleural effusions greater than 1 cm on a lateral decubitus film warranted sampling [6]. However, lateral decubitus films are rarely performed anymore due to the widespread availability of ultrasound and computer tomography scanning. With that in consideration, the most recent guidelines suggest that diagnostic thoracentesis should be performed on all parapneumonic effusions with a pleural fluid thickness of >1 cm on chest X-ray or >2 cm on chest CT [4].

Ultrasound should be used to confirm the presence of pleural effusion and ultimately to guide diagnostic thoracentesis. Upon sampling, the presence of pus, positive Gram stain, or culture establishes the diagnosis of empyema and should prompt drainage via tube thoracostomy [4]. The absence of pus should prompt further fluid analysis with pH measurement within one hour of sampling using an arterial blood gas analyzer with additional measurement of pleural fluid glucose, pleural fluid lactate dehydrogenase, pleural fluid cell count, culture, and Gram stain at minimum [4]. Traditional predictors of a complicated clinical course using pleural fluid analysis include a pH < 7.20, pleural fluid glucose of <40 mg/dL, pleural fluid LDH >1000 IU/L, and positive Gram stain or culture, all of which should prompt drainage via tube thoracotomy [4]. Complex effusions with septations do raise certain considerations in that isolated pockets may be sampled and may be misleading on a biochemical basis if used as the sole factor in deciding to proceed with pleural space drainage. Specifically, Maskell et al. compared biochemical findings in different loculations of patients presenting with parapneumonic effusion and specifically noted variability in pH that would have otherwise guided clinicians away from definitive drainage had other samples not been taken [39].

More recently, there has been research into alternative markers of pleural infection. Specifically, pleural fluid levels of soluble urokinase plasminogen activator receptor (SuPAR) have garnered attention as a potential novel biomarker for complicated pleural space infections. Arnold and colleagues found that SuPAR levels were significantly higher in loculated vs. non-loculated effusions and that SuPAR levels more accurately predicted a complicated clinical course than traditional markers such as pH, glucose, and LDH. Specifically, levels greater than 35 ng/mL were 83% sensitive and 92% specific for eventual tube thoracostomy, and levels > 65 ng/mL were 94% sensitive and 84% specific for eventual intrapleural fibrinolytics or thoracic surgery [40].

Additional pleural fluid markers have been evaluated and include procalcitonin, calprotectin, presepsin, and C-reactive protein. Pleural procalcitonin levels > 0.25 ng/mL specifically were noted to have a sensitivity of 77.78% and a specificity of 74.14% for distinguishing infectious pleural effusion from non-infectious pleural effusion [41]. Additionally, pleural procalcitonin at a level > 0.25 ng/mL was associated with a longer total length of hospital stay (17.0 vs. 8.7 days, 95% CI: −12.5 to −4.0, *p* < 0.001) and a higher rate of complication (38% vs. 0%, *p* = 0.004) in a retrospective analysis of 38 patients who received surgery for complicated pleural space infections [42]. Pleural fluid calprotectin levels have also been studied in exudative pleural effusions and have specifically been noted to be significantly lower in exudative pleural effusions secondary to malignancy as compared with exudative effusions caused by infection [43]. Presepsin, also known as Soluble CD14 Subtype, has recently gained interest as a potential biomarker for sepsis, and Presepsin levels in pleural fluid were shown to be significantly higher in non-tuberculosis pleural infections and empyema as compared with other exudative effusions and transudates [44,45]. Additionally, Huang et al. recently published results indicating that pleural fluid levels of Nicotinamide Phosphoribosyltransferase (NAMPT) were significantly elevated in effusions due to pleural infections and tuberculosis pleural effusions as compared with malignant pleural effusions and transudative causes [46].

Finally, pleural fluid C-reactive protein levels have been extensively studied and have been shown to be useful in distinguishing pleural infection and empyema from other non-infectious exudative effusions. Specifically, a pleural fluid CRP level of less than 13.8 mg/L has a high negative predictive value for distinguishing pleural infections from effusions secondary to malignancy, heart failure, or lung transplant [47]. Additionally, higher levels of pleural fluid CRP have shown to be useful in the diagnosis of pleural infection and in predicting which pleural infections and parapneumonic effusions will require mechanical drainage. Specifically, a pleural fluid CRP level of >45 mg/L in the setting of a 50% or greater neutrophil-predominant pleural effusion had a positive likelihood ratio of 7.7 for diagnosis of parapneumonic effusion/pleural infection and was more sensitive and specific than traditional markers such as glucose, LDH, and pleural fluid pH [48]. Additionally, pleural fluid CRP levels >100 mg/L with a pleural fluid glucose of <60 mg/dL were found to have a positive likelihood ratio of 15.5 for requiring chest tube drainage [48]. Kogan et al. reported similar findings, specifically noting that pleural fluid CRP levels of >90.5 mg/L were 57.6% sensitive but 83.8% specific for distinguishing complicated from uncomplicated parapneumonic effusion. They also evaluated the serum-to-pleural fluid CRP gradient and ratio. While the ratio was not found to be useful, a gradient greater than 142 mg/L (serum CRP minus pleural fluid CRP) was found to be 90% sensitive and 86% specific, with a positive likelihood ratio of 55.5 for distinguishing complicated vs. uncomplicated parapneumonic effusion [49]. Table 2 displays a summary of novel markers for pleural infection and the significance of their findings. 

Serum CRP levels may be valuable for monitoring response to therapy and resolution of pleural space infections [15]. However, the utility of following CRP levels after procedural intervention is less useful. Medeiros et al. analyzed CRP levels after surgical decortication and did not find any association with the ultimate outcome [50]. Additionally, CRP levels have been noted to rise following surgery, peaking between 3 and 6 days post-decortication with an eventual decline to less than 50 mg/L by post-operative day twelve [51]. This is in line with other studies that found similar patterns [5].

## 10. Antibiotic Therapy

As with any infection, source control with prompt initiation of antibiotics is paramount for infection control and patient recovery, and pleural infections are no different. Specific factors that should be considered are the clinical history of the patient (and any risk factors for resistant organisms or hospital-acquired organisms), local antibiotic resistance pattern, pharmacologic characteristics of the potential antibiotics, ability to penetrate the pleural space, and local antibiotic institutional stewardship [1,4]. Specifically, penicillin, ceftriaxone, metronidazole, and clindamycin have all shown good penetration into the pleural space [15].

For patients with community-acquired pleural empyema with a low risk of methicillin-resistant organisms or other resistant Gram-negative organisms, reasonable empiric antibiotic regimens include a non-pseudomonal second-generation cephalosporin, a third-generation cephalosporin, or an aminopenicillin with a B-lactamase inhibitor [1,4]. Consideration of anaerobic coverage is also somewhat unique when treating pleural space infections in that the addition of anaerobic coverage with metronidazole or clindamycin is generally advisable [1,4,52]. This contrasts treatment for community-acquired pneumonia (CAP) as the recently published joint American Thoracic Society–Infectious Disease Society of America guidelines do not recommend routine anaerobic coverage [52]. Coverage for atypical organisms with macrolide therapy is generally also not required and is not routinely advised; however, empyema secondary to legionella, which has rarely been reported, should be treated with a macrolide antibiotic [1]. Aminoglycosides have no role in the treatment of empyema as they penetrate poorly into the pleural space and may be inactive in the generally acidic environment of infected pleural fluid [1,4]. 

Hospital-acquired pleural space infections raise different considerations for antibiotic selection and may result from nosocomial pneumonia or surgery. Specifically, antibiotic therapy for patients with these risk factors should be expanded to cover MRSA and Pseudomonas. Specifically, Staphylococcus aureus will comprise approximately 50% of positive pleural fluid cultures in patients with hospital-acquired pleural infections, with MRSA representing about two-thirds of these cases and the remainder being Gram-negative organisms (predominantly E. coli, Enterobacter, and Pseudomonas) [1]. Data for direct inoculation of antibiotic therapy into the pleural space is lacking and is currently not supported by any guidelines, with one possible exception being in specific post-surgical scenarios [4,8,15].

De-escalation of antibiotic therapy with eventual transition to oral antibiotics is a tenant of good antimicrobial stewardship. With that, transition to oral therapy is indicated when the isolated organism is susceptible to oral therapies, source control has been achieved, and there is objective improvement in the patient’s clinical status [4]. Duration of therapy is not well defined but should be at least 2–3 weeks and may be as long as 6 weeks for empyema. However, the duration ultimately should be guided by response to therapy and adequacy of drainage as demonstrated by clinical resolution of symptoms, radiographic improvement and/or resolution, and improvement in laboratory markers of infection such as CRP [1,4].

## 11. Intrapleural Medical Therapies

A review of the treatment of pleural space infections is not complete without a discussion of the role of Intrapleural Enzymatic Therapy. While this was first described in 1948 by Drs. William Tillett and Sol Sherry, it was not until the publication of the second Multicenter Intrapleural Sepsis Trial (MIST2) in 2011 that a definite benefit was demonstrated. Specifically, the MSIT2 trial was a double-blind, double-dummy factorial randomized trial conducted at 11 centers in the United Kingdom from 2005 to 2008 that compared various combinations of tissue plasminogen activator (t-PA) and deoxyribonuclease (DNase) vs. placebo in the treatment of pleural space infections. Inclusion criteria were adult patients with clinical evidence of pleural space infection (fever, elevated inflammatory markers, leukocytosis) and pleural fluid that was macroscopically purulent, positive for bacteria on culture, positive on Gram staining for bacteria, or pleural fluid with a pH of less than 7.20. 

These patients were then randomized to one of four groups: t-PA and DNase, DNase and placebo, t-PA and placebo, or double placebo. Primary outcomes were the change in pleural opacity on chest radiograph at day 7 vs. day 1, along with various secondary endpoints. Ultimately, the t-PA and DNase group showed a statistically significant decrease (−29.5%) in pleural opacity from day 1 to day 7 vs. placebo. Also notable was a decrease in referral to surgery at 3 months in the t-PA and DNase group vs. placebo (OR 0.27, confidence interval 0.03 to 0.87, *p*-value 0.03). Mortality rates were similar across all four groups, however [53]. Because the MIST2 trial combined tPA (a fibrinolytic) with DNase (an endonuclease that breaks down extracellular DNA, thereby decreasing viscosity), the term Intrapleural Enzyme Therapy (IEP) more accurately describes this treatment and is now favored over Intrapleural Fibrinolytic Therapy. 

In 2014, Piccolo et al. performed a retrospective observational study examining the efficacy of t-PA and DNase use in patients with pleural infection in a cohort of patients combined from centers in Australia, New Zealand, and the United Kingdom. In this study, 92.3% of patients who received t-PA and DNase were successfully treated without surgery, with only 8 of the 107 patients requiring surgery, all of whom survived to 90 days. Three deaths were observed before the 30-day mark [54]. While the initial MIST2 study and subsequent study by Piccolo and colleagues used sequential therapy, more recently, Majid et al. showed that concurrent administration of tPA and DNase was effective with an overall treatment success rate of 90.4%. Additionally, this therapy was relatively safe, with a bleeding rate requiring transfusion of only 5.4%, which was similar to the data reported in the MIST2 trial (6%) but higher than that reported by Piccolo (1.8%) [55]. 

It is also worth noting that while the MIST2 study examined six total doses of t-PA and DNase, alternative dosing regimens have been evaluated and have been generally safe. Specifically, extending therapy beyond six doses has not been shown to increase the rate of complications or eventual need for surgery and was not associated with a difference in outcomes [56]. Additionally, once-daily therapy with intrapleural fibrinolytic therapy was shown to be similarly effective as compared with traditional dosing, with an added benefit of an estimated cost savings of USD 2657.14 per three-day course [57]. Similarly, dose reduction to 5 mg of alteplase (as opposed to 10 mg) has been studied due to concerns about bleeding and in an effort to reduce costs and has been shown to be effective as well [58]. In line with this, a recent retrospective cohort study found that intrapleural fibrinolytic therapy, as prescribed by the original MIST2 study, was only utilized in 52% of cases for which intrapleural therapy was used, suggesting substantial physician variability in practice patterns [59]. Contraindications to intrapleural fibrinolytic therapy include coagulopathy, concurrent anticoagulation therapy, allergy, or hypersensitivity to the medications. The presence of bronchopleural fistula is also a contraindication [55].

## 12. Procedural Management

Overall, there is little quality evidence to support either medical therapy with tube thoracotomy or surgery as a superior first-line option in managing adults with complicated pleural space infections. A comprehensive meta-analysis performed in 2017 found only eight randomized controlled trials since 1946 that examined this question, and of these eight trials, six were trials in children, with only two examining medical vs. surgical therapy for empyema in adults. Notable conclusions from this analysis were that VATS showed a statistically significant reduction in hospital length of stay (−2.52 days) vs. medical therapy for adults; however, there were no data to show improved mortality or any differences in the rate of procedural complications [60]. More recently, Wilshire et al. compared initial surgery vs. fibrinolytics through retrospective cohort analysis and did not find a mortality benefit of either management strategy. However, the results were in line with prior studies showing initial surgery to be associated with a shorter overall hospital length of stay, shorter duration of chest tube, lower rates of additional treatments and treatment failures, and lower risk of readmission, all of which met statistical significance [59].

While the published guidelines do offer a consensus regarding the necessity for pleural drainage in complicated parapneumonic effusions and empyema, the specific recommendations for further procedural approaches are less clear. The European Journal of Cardiothoracic Surgery in 2015 stated that surgical debridement or decortication were superior to tube thoracotomy for stage 2 and 3 empyema; however, this contrasts the findings of the meta-analysis performed by Redden et al. in 2017, which showed no mortality benefit [8,60]. Similarly, the 2017 American Academy of Thoracic Surgery guidelines recommend tube thoracotomy for initial management and VATS as the preferred surgical procedure in all patients with stage II or III empyema “requiring surgical intervention”. However, little guidance is offered for what objective data should prompt surgical intervention aside from failing to achieve complete pleural drainage or failure to improve clinically, which should prompt consideration for more aggressive therapy to include intrapleural enzyme therapy or VATS [4]. The 2010 British Thoracic Society offers perhaps the most granular guidance on when surgery should be considered, stating that patients with persistent sepsis that fails to resolve in 5–7 days, in association with non-draining fluid in a pleural infection who are on appropriate antibiotic therapy should prompt consideration for surgery [1]. More recently, the ERS also proposed goals of surgery by day 10 if required, with surgical consultation on day 3, with factors meriting consideration being ongoing sepsis, failure of radiographic resolution, or clinical deterioration [15]. The newly drafted but unpublished BTS guidelines from June 2022 do not appear to definitively address this question [61].

Recent studies have also reported variable survival based on the microbiology of a pleural space infection. Kanellakis et al. specifically found that the presence of S. aureus was associated with significantly lower 12-month survival as compared with other organisms vs. infections where S. aureus was absent (hazard ratio of 5.80, range 2.37–14.21, *p* < 0.0001). Additionally, the dominance of either S. aureus or Enterobacter in polymicrobial infections was associated with worse survival (HR 3.97, range 1.20–13.08, *p* = 0.024, HR 2.26, 1.03–4.93, *p* = 0.041, respectively). The presence of anaerobic organisms or organisms of the Staph anginous group was associated with improved survival (HR 0.46, CI 0.24–0.86, *p* = 0.015, 0.043, 0.19–0.97, *p* = 0.043, respectively) [62]. However, using microbiologic data to prospectively guide the clinical course has obvious limitations, as pleural fluid cultures are only positive in approximately half of patients. However, pleural biopsies have recently been shown to be useful in increasing this yield [63]. 

More recently, medical thoracoscopy has shown promise in potentially bridging the gap between tube thoracostomy and surgery. The advantages are that this procedure can be performed without general anesthesia using only local anesthesia with moderate sedation through a single port placed with the patient in the lateral decubitus position, and thus, may be better tolerated by patients who are poor surgical candidates. A recent meta-analysis found that medical thoracoscopy was safe and effective, with an 85% pooled success rate [64]. Medical thoracoscopy was also found to be associated with a shorter length of hospital stay post-intervention as compared with intrapleural fibrinolytic therapy (2 days vs. 4 days, *p* = 0.026); however, there was no statistically significant difference in success rate [65]. Perhaps most attractive is that pooled meta-analysis data have suggested improved success through combining medical thoracoscopy followed by intrapleural enzyme therapy, as medical thoracoscopy allows for targeted debridement of adhesions and precise placement of chest tubes [64]. Accordingly, medical thoracoscopy combined with intrapleural fibrinolytic therapy may be an emerging option for patients without prompt resolution of pleural infections who are not good surgical candidates; however, randomized clinical data is lacking. 

Saline irrigation in the setting of pleural infection has been another recent area of research. Hooper et al. randomized patients with pleural infection who failed to drain after initial chest tube to either standard care with chest tube flushes or three times daily infusion of 250 cc of normal saline into the affected pleural cavity. Their results showed a statistically significant reduction in pleural collection when compared with normal therapy (32% vs. 15%, *p* < 0.04), a decreased need for eventual surgery (2/18 vs. 8/18, *p* = 0.03), and were well tolerated with few side effects [66]. Similarly, Porcel et al. performed a retrospective analysis of parapneumonic effusions treated with a forceful injection of various amounts of normal saline in conjunction with intrapleural urokinase. While retrospective, this study demonstrated the following statistically significant outcomes: intrapleural saline increased the total amount of fluid drained, reduced the fibrinolytic treatment duration, and decreased the time from chest tube insertion to removal [67]. More recently, Guinde et al. also retrospectively evaluated normal saline irrigation via gravity in variable amounts and found that only four of thirty patients required eventual surgery, concluding that intrapleural normal saline was safe and effective for pleural space infections [68]. Combined, normal saline irrigation may be an attractive, simple, cost-effective, and underutilized modality for the initial treatment of pleural space infections. Combining data from the aforementioned studies by Hooper, Porcel, and Guinde for patients treated with normal saline irrigation shows a 3-month mortality of 8.45% (6/71), a surgical referral rate of 9.86% (7/71), and a hospital length of stay of 11.1 days (weighted across all studies). Comparing this to the DNase/tPA arm of MIST2 is notable for a similar 3-month mortality (4/48, 8.33%) and hospital length of stay (11.8 days), although the decreased rate of referral to surgery is notable (2/48, 4.17%).

Lastly, therapeutic thoracentesis with repeat thoracentesis has been evaluated as well. Specifically, repeat thoracentesis was noted to have a success rate of 76% in complicated parapneumonic effusions and empyema and may offer benefits in terms of preserving patient mobility and potentially facilitating outpatient treatment [15]. This may be an option for low-risk patients with small effusions but is not supported by any guidelines for complicated pleural infections. Overall, these data may best support actively monitoring a low-risk patient who presented with a small effusion that was drained to completion and later was found to be complicated by laboratory or microbiologic analysis, whereby the prior drainage would make subsequent placement of a chest tube technically difficult. 

## 13. Future Research

Ultimately, pleural space infections, parapneumonic effusions, and empyema as clinical entities are heterogenous in their presentation and severity. Additionally, patient-specific factors and clinical context play strongly into clinical decision-making, especially concerning procedural selection and timing. With that, current research endeavors will hopefully provide data to optimize future pleural infection management decisions. 

Two notable trials awaiting publication are the SPIRIT trial and the MIST3 trial. The SPIRIT trial seeks to evaluate medical pleuroscopy as an intervention in pleural infection and was reportedly due to release its results in late 2021, but it has not yet been published [69]. MIST3 seeks to randomize patients with pleural infections to upfront surgery, early intrapleural enzymatic therapy, or standard care and has reportedly completed enrollment but has not yet published results [70]. 

Some notable trials that are currently ongoing are the FIVERVATS study which is seeking to assess whether VATS or tube thoracotomy with intrapleural enzyme therapy is more effective in treating stage II and III complicated parapneumonic effusions/pleural infections [71]. Similarly, Chung et al. seek to compare outcomes in the fibrinopurulent phase of pleural infection based on initial treatment with VATS or IR-guided chest tube with fibrinolytic therapy (results anticipated in 2024) [72]. This trial will conclude enrollment in September 2023, with one-year follow-up data available in September 2024, and may help better delineate which procedure is most beneficial at which stage. 

Additionally, steroid therapy has been proposed as an adjunctive treatment for curbing the inflammatory response and progression of parapneumonic effusion. With that, the Steroids Therapy and Outcomes in Parapneumonic Pleural Effusions (STOPPE) trial is currently ongoing and aims to assess whether systemic steroids have a benefit in outcomes in patients with parapneumonic effusions [73]. Treatment of parapneumonic effusion with intrapleural 2% povidone-iodine solution is also an area of active research, with reported completion of a phase one trial and a phase two trial currently undergoing recruitment. These studies seek to assess whether intrapleural povidone-iodine irrigation has a benefit over placebo irrigation with normal saline [74,75]. No results are yet available for the phase 1 trial; however, phase 2 results are anticipated in 2024.

Improving the microbiologic diagnostic yield with next-generation sequencing is another area of active research and may help guide clinical decision-making, as variable mortality has been noted with different organisms [62]. With that, ongoing efforts by Chan et al. are currently underway to add additional data regarding advanced techniques to microbiologically diagnose specific pleural space infections; however, results are not anticipated until 2027 [76]. Additionally, pleural biopsies may be an emerging future tool to increase the microbiologic yield, as the AUDIO study showed that adding pleural biopsies to pleural fluid and blood cultures increased diagnostic yield by 25% [63].

## 14. Conclusions

In conclusion, pleural infections represent a diverse clinical spectrum of disease that varies from early exudative effusions (stage 1) that will generally resolve with antibiotics alone to advanced complicated effusions that require mechanical procedural drainage. As with any infection, tenants of good care include prompt diagnosis and source control when indicated and prompt administration of antibiotic therapy. While not incorporated into any current guidelines, recent advances in pleural biochemical and microbiological diagnostics and radiographic imaging may ultimately prove useful in predicting which patients will require advanced therapies and will be a focused area of research in the coming decade. Additionally, further research into procedural management strategies, especially relating to the phase of the infection, will help clarify when advanced intervention should be performed and in which patients. 

## Figures and Tables

**Table 1:** Definitions of terms in pleural infections.

Pleural Infection
Bacterial or Fungal entry and replication in the pleural space
Parapneumonic Effusion
Any pleural effusion associated with pneumonia or lung abscess
Uncomplicated Parapneumonic Effusion
A parapneumonic effusion that will resolve without mechanical intervention, and with only antibiotics
Complicated Parapneumonic Effusion or Complicated Pleural Infection
A pleural infection that will require mechanical drainage for resolution
Classically, pleural fluid: pH < 7.20, Glucose < 40 mg/dL, LDH > 1000 IU/L
Empyema
Pus in the pleural space (a thick, white-yellow, viscous fluid), positive pleural fluid gram stain or culture
Complex Pleural Effusion
A physical description of a pleural effusion, often with septations and loculations. Not specific to plerual infections.

**Table 2:** Summary of novel markers for pleural infection with their associated findings.

Pleural Fluid SuPAR
Level > 35 ng/mL is 83% Sensitive, 92% Specific for eventual tube thoracostomy
Level > 65 ng/mL is 94% Sensitive, 84% Specific for eventual IPE therapy or surgery.
Pleural Procalcitonin
Level > 25 ng/mL is 78% Sensitive, 74% Specific for distinguishing Infectious vs Non-Infectious Pleural Effusion
Pleural Calprotectin
Significanty lower in exudates caused by infection as compared with malignancy.
Pleural Fluid Presepsin
Significantly higher in non-TB parapneumonic effusion and empyema compared with other exudates.
Pleural NAMPT
Significantly elevated in parapneumonic effusion and TB pleural effusions as compared with malignant effusions.
Pleural Fluid C-Reactive protein
Level less than 13.8 mg/L has a high NPV for parapneumonic.
Level > 45 mg/L with 50% or more Neutrophils on PF differential has a positive LR of 7.7 for parapneumonic effusion.
Level >100 mg/L with glucose <60 mg/dL has a positive LR or 15.5 for requring tube thoracotomy.
Serum to Pleural Fluid CRP Gradient of >142 mg/L was 90% Sensitive, 86% specific with psitive LR of 55.5 for distinguishing compliated from uncomplicated parapneumonic effusion.

## Data Availability

No new data were created or analyzed in this study. Data sharing is not applicable to this article.
